# The Medical Formulary: Being a Collection of Prescriptions, &c.

**Published:** 1864-03

**Authors:** 


### Book Notices.

THE MEDICAL FORMULARY: Being a Collection of Prescriptions derived from the Writings and Practice of many of the most Eminent Physicians in America and Europe. Together with the usual Dietic preparations and Antidotes for Poisons. To which is added an Appendix on the Endermic use of Medicines, and on the use of Ether and Chloroform. The whole accompanied with a few brief Pharmaceutical and Medical Observations. By BENJAMIN ELLIS, M.D., late Professor of Materia Medica and Pharmacy in the Philadelphia College of Pharmacy. Eleventh Edition, Carefully Revised and much Extended, by ROBERT P. THOMAS, M.D., Professor of Materia Medica in the Philadelphia College of Pharmacy. Philadelphia: BLANCHARD & LEA. 1864.

This is a neatly published volume of 341 pages, the contents of which are fully indicated by the above lengthy title. The work has long been before the profession, and its merits are well known. The present edition contains many valuable additions, and will be found to be an exceedingly convenient and useful volume for reference by the medical practitioner.

For sale by W. B. KEEN, 148 Lake street, Chicago.

